# Decentralizing Pulmonary Screening for China’s Aging Population: From Hospitals to Communities

**DOI:** 10.14336/AD.2025.1027

**Published:** 2026-08-20

**Authors:** Leiwen Fu, Wei Shu, Yuxian Sun, Liang Li, Jian Du

**Affiliations:** ^1^Beijing Chest Hospital, Capital Medical University, Beijing Tuberculosis and Thoracic Tumor Research Institute, Beijing, China.

**Keywords:** aging population, chronic respiratory diseases, pulmonary screening, community-based care, low-dose CT (LDCT), health disparities

## Abstract

China’s aging population faces a growing burden of chronic respiratory diseases, straining the public health system. Despite the Healthy China 2030 plan’s emphasis on prevention and early detection, systemic barriers, such as low public awareness, urban-rural disparities, fragmented screening models, and uneven specialist distribution, hinder effective pulmonary care for older adults. To address these challenges, a shift toward community-based strategies, including mobile low-dose CT (LDCT) screening units, is proposed. These units improve accessibility, especially in rural areas, while AI integration enhances diagnostic accuracy and telemedicine bridges specialist gaps. Strengthening local healthcare capacity through training, policy support, and coordinated care among community health stations, hospitals, and the CDC is critical. Community engagement further boosts participation and follow-up. Success will be measured by early diagnosis rates, reduced disparities, and cost-effectiveness. Aligning with Healthy China 2030, this integrated approach promises equitable, sustainable respiratory care for aging populations.

China rapidly transitions to an aging society; its public health system is increasingly burdened by the growing prevalence of chronic respiratory diseases among older adults [1]. The Healthy China 2030 plan (www.gov.cn/zhengce/202203/content_3635233.htm) emphasizes the need for enhanced health management for the aging population, with a focus on disease prevention and early detection, particularly for chronic respiratory conditions [2]. Diseases such as lung cancer, chronic obstructive pulmonary disease, and tuberculosis represent a significant portion of the national disease burden.

Several systemic barriers hinder effective pulmonary screening for elderly populations in China. First, limited public awareness and misconceptions about respiratory illnesses lead to poor engagement with preventive measures and screenings [3]. Second, accessibility remains a significant challenge due to pronounced urban-rural disparities, mobility limitations, financial burdens, and medical anxiety, compounded by difficulties navigating complex hospital systems [4, 5]. Additionally, the current fragmented model for respiratory care often focuses primarily on individual diseases rather than employing a coordinated strategy that links prevention, screening, and treatment [6]. Lastly, the uneven regional distribution of medical expertise, with specialists concentrated in urban centers, exacerbates diagnostic and healthcare inequalities [7]. These challenges align with the Healthy China 2030 plan's objective to reduce health disparities between urban and rural areas and improve healthcare access for older adults, particularly in under-resourced regions [2]. A paradigm shift towards community-based preventive strategies is urgently required.

A particularly promising solution is the deployment of mobile low-dose computed tomography (LDCT) screening units, which bring essential diagnostic services directly into communities [8]. In the Healthy Aging Initiative in China's 14th Five-Year Plan (www.gov.cn/gongbao/content/2022/content_5692863.htm), it is emphasized that the capacity of basic public health services to promote elderly health should be strengthened. Additionally, the plan calls for innovation in continuous service models to enhance healthcare accessibility for the elderly population. Mobile units offer a cost-effective and flexible alternative to stationary LDCT scanners, particularly in resource-limited settings. By rotating among multiple locations, a single mobile LDCT unit can efficiently serve numerous communities, maximizing equipment utilization. This approach significantly improves accessibility for elderly patients who face transportation challenges or mobility limitations, enabling convenient and simultaneous screening for multiple respiratory conditions. Combining prevention with simultaneous screening for multiple respiratory conditions holds substantial promise, particularly for tuberculosis control, given China's status as a high-burden nation [9].

Integrating artificial intelligence (AI) with mobile CT screening significantly enhances program efficiency and diagnostic accuracy [10]. AI-powered platforms quickly identify abnormalities, while telemedicine networks seamlessly connect local screenings to specialist consultations, eliminating the necessity for long-distance patient travel and bridging the gap between community and specialized healthcare services. The integration of AI with CT is also identified as one of the recommended application scenarios in the Reference Guidelines for Artificial Intelligence Application Scenarios in the Health Industry issued by the National Health Commission (www.nhc.gov.cn/guihuaxxs/c100133/202411/3dee425b8dc34f739d63483c4e5c334c.shtml). This integration is demonstrated to significantly enhance both the operational efficiency of screening programs and the diagnostic precision. For example, Beijing Chest Hospital has deployed mobile LDCT units to conduct free screening for lung cancer and pulmonary infections in communities across various regions of China (www.beijing.gov.cn/xwzx_20031/jcdt/202411/t202411203946205.html). By integrating AI-assisted diagnostics and remote consultations with imaging experts, the quality of healthcare services for residents in remote areas has been significantly enhanced. Among the 46,000 individuals screened, more than 800 high-risk pulmonary nodules and over 700 suspected cases of pulmonary tuberculosis were detected and referred for further confirmation.

Effective implementation of community-based pulmonary screening requires seamless coordination between community health stations, hospitals, and the Center for Disease Control and Prevention (CDC). Community health stations, as the frontline of screening, not only identify potential cases but also play a crucial role in health education, raising awareness about respiratory diseases and the importance of early detection, in collaboration with the CDC. The CDC ensures that health education efforts align with national health priorities and provides guidance on disease prevention. Following screening, hospitals are responsible for specialized diagnosis and treatment, while community health stations and CDC remain involved in-patient follow-up and care. Community health stations monitor patients' progress, educate on treatment adherence, and offer rehabilitation support, while hospitals handle more complex medical care. The CDC ensures that all care is consistent with national health policies and provides additional resources for managing chronic conditions. This screening, referral and feedback mechanism ensures continuity of care across healthcare levels, facilitates timely clinical interventions, and supports the sustainability of screening programs by integrating community-based prevention and early detection with specialized diagnosis, treatment, and longitudinal follow-up.

Strengthening the capabilities of local medical personnel through systematic training and telemedicine is equally critical [11]. Continuous professional education and remote mentoring empower primary care providers and community health workers, improving their respiratory care skills. Furthermore, establishing evidence-based healthcare policies that support service coordination across care levels and ensure sustainable financing is essential. In addition to these technological and policy innovations, active community engagement is essential. Community health workers play a crucial role in motivating elderly individuals, facilitating higher participation rates and consistent follow-up care. China is exploring a comprehensive prevention, screening, diagnosis, treatment, and care service model for elderly, based on community-level service centers.

The translational value of community-based pulmonary screening will be assessed through measurable outcomes including increased early-stage diagnosis rates, improved follow-up adherence, and reduced urban-rural disparities in screening participation. Additional evaluation metrics include smoking cessation rates, diagnostic accuracy, cost-effectiveness, and improvements in quality of life. These indicators provide policymakers with an evidence base for program optimization and sustainable implementation across diverse healthcare settings.

Although the considerable potential of mobile AI-assisted pulmonary screening, its widespread implementation faces several critical challenges that merit careful deliberation. Foremost among these is the issue of diagnostic accuracy, where false-positive results may precipitate unnecessary patient anxiety and invasive follow-up procedures, while false-negatives could delay essential interventions [12]. The specter of overdiagnosis looms particularly large in elderly populations, potentially leading to overtreatment of indolent conditions [13]. Equally pressing are data governance concerns, as the integration of telemedicine networks with AI platforms necessitates stringent adherence to cybersecurity protocols and robust encryption standards to safeguard sensitive health information. Geographic disparities in infrastructure present another barrier, with rural and resource-limited regions often lacking the stable electricity and broadband connectivity required for seamless operation. Perhaps most fundamentally, ethical imperatives demand rigorous attention to informed consent processes that adequately disclose the role of AI in diagnostic decision-making [14]. Ensuring the long-term viability of such programs will require innovative financing mechanisms, including public-private partnerships and tiered reimbursement models, coupled with sustained governmental commitment to workforce training and technological maintenance.

In summanry, shifting from hospital-centered model to community driven preventive approach is fully alligned a people-centered principle and enable effective addressing the respiratory health challenges posted by China’s aging population. Community-based pulmonary screening models that integrate mobile imaging, AI-assisted diagnostics, and strengthened primary care are not only critical with feasible in China but are also highly applicable to other low- and middle-income countries confronting similar challenges of aging populations and increasing respiratory disease burdens. These efforts will significantly improve pulmonary screening outcomes, ensuring equitable, sustainable, and high-quality respiratory healthcare for China's aging communities.
